# Combining Attractants and Larvicides in Biodegradable Matrices for Sustainable Mosquito Vector Control

**DOI:** 10.1371/journal.pntd.0005043

**Published:** 2016-10-21

**Authors:** Dirk Louis P. Schorkopf, Christos G. Spanoudis, Leonard E. G. Mboera, Agenor Mafra-Neto, Rickard Ignell, Teun Dekker

**Affiliations:** 1 Swedish University of Agricultural Sciences, Unit of Chemical Ecology, Department of Plant Protection Biology, Alnarp, Sweden; 2 Aristotle University of Thessaloniki, Faculty of Agriculture, Laboratory of Applied Zoology and Parasitology, Thessaloniki, Greece; 3 National Institute for Medical Research, Dar es Salaam, Tanzania; 4 ISCA Technologies Inc., Riverside, California, United States of America; University of Florida, UNITED STATES

## Abstract

**Background:**

There is a global need for cost-effective and environmentally friendly tools for control of mosquitoes and mosquito-borne diseases. One potential way to achieve this is to combine already available tools to gain synergistic effects to reduce vector mosquito populations. Another possible way to improve mosquito control is to extend the active period of a given control agent, enabling less frequent applications and consequently, more efficient and longer lasting vector population suppression.

**Methodology/principal findings:**

We investigated the potential of biodegradable wax emulsions to improve the performance of semiochemical attractants for gravid female culicine vectors of disease, as well as to achieve more effective control of their aquatic larval offspring. As an attractant for gravid females, we selected acetoxy hexadecanolide (AHD), the *Culex* oviposition pheromone. As toxicant for mosquito larvae, we chose the biological larvicides *Bacillus thuringiensis israelensis* (*Bti*) and *Bacillus sphaericus* (*Bs*). These attractant and larvicidal agents were incorporated, separately and in combination, into a biodegradable wax emulsion, a commercially available product called SPLAT (Specialized Pheromone & Lure Application Technology) and SPLATbac, which contains 8.33% *Bti* and 8.33% *Bs*. Wax emulsions were applied to water surfaces as buoyant pellets of 20 mg each. Dose-mortality analyses of *Culex quinquefasciatus* Say larvae demonstrated that a single 20 mg pellet of a 10^−1^ dilution of SPLATbac in a larval tray containing 1 L of water caused 100% mortality of neonate (1^st^ instar) larvae for at least five weeks after application. Mortality of 3^rd^ instar larvae remained equally high with SPLATbac dilutions down to 10^−2^ for over two weeks post application. Subsequently, AHD was added to SPLAT (emulsion only, without *Bs* or *Bti*) to attract gravid females (SPLATahd), or together with biological larvicides to attract ovipositing females and kill emerging larvae (SPLATbacAHD, 10^−1^ dilution) in both laboratory and semi-field settings. The formulations containing AHD, irrespective of presence of larvicides, were strongly preferred as an oviposition substrate by gravid female mosquitoes over controls for more than two weeks post application. Experiments conducted under semi-field settings (large screened greenhouse, emulating field conditions) confirmed the results obtained in the laboratory. The combination of attractant and larvicidal agents in a single formulation resulted in a substantial increase in larval mosquito mortality when compared to formulations containing the larvicide agents alone.

**Conclusions/significance:**

Collectively, our data demonstrate the potential for the effective use of wax emulsions as slow release matrices for mosquito attractants and control agents. The results indicate that the combination of an oviposition attractant with larvicides could synergize the control of mosquito disease vectors, specifically *Cx*. *quinquefasciatus*, a nuisance pest and circumtropical vector of lymphatic filariasis and encephalitis.

## Author Summary

Traditionally, a key intervention in mosquito control is the use of insecticides against the adult stage. However, various factors limit the long-term use of these control methods, including the development of insecticide resistance, changes in mosquito biting behaviour, and concerns regarding potential negative impacts of insecticides on the environment. There is therefore a need for alternative management strategies, such as those that target aquatic life stages of mosquitoes. The objective of this study was to investigate the potential of biodegradable wax emulsions such as SPLAT for use in attracting gravid females and control of aquatic stages of culicine vectors. *Culex* mosquito oviposition pheromone (acetoxy hexadecanolide, AHD) was selected as an attractant, and *Bacillus thuringiensis israelensis* (*Bti*) and *Bacillus sphaericus* (*Bs*) were used as control agents. Buoyant 20 mg pellets, created by drying SPLAT dollops prior to application, were applied to water surfaces. Dose-mortality analyses of *Cx*. *quinquefasciatus* larvae demonstrated that one single pellet caused 100% mortality of first instar larvae for at least five weeks post application. Mortality of 3^rd^ instar larvae remained equally high even at 10^−2^ dilutions for over two weeks post application. In addition, AHD was embedded in SPLAT to either attract gravid females (SPLATahd) or to first attract gravid females to oviposit and then to kill the resulting larval offspring (SPLATbacAHD, 10^−1^ dilution) in both laboratory and semi-field settings. The wax matrix containing AHD, with or without *Bti* and *Bs*, was strongly preferred as an oviposition substrate over controls for over two weeks post application. Both laboratory and semi-field experiments showed a marked increase in larval mortality effects when a semiochemical attractant and larvicides were combined, compared to matrices containing larvicides alone. These findings indicate the potential for using wax emulsions such as SPLAT as a slow release matrix for mosquito attractants and control agents; and that the combination could synergize the control of *Cx*. *quinquefasciatus*.

## Introduction

Mosquitoes transmit several serious pathogens in the tropics. Mosquito population control interventions are therefore frequently implemented to keep the diseases at bay, which are caused by those pathogens. The most targeted mosquito species include members of the mosquito genera *Anopheles* (vectors of malaria,[1]), *Aedes* (vectors of dengue, yellow fever virus, chikugunya, Zika, Rift Valley fever and many other arboviruses,[2, 3]) and *Culex* (vectors of lymphatic filariasis and West Nile virus,[4, 5]). Control measures often target adults and may take the form of indoor residual spraying [1, 6, 7], space spraying [3, 8] and insecticide treated materials such as curtains and bed nets [9–13]. However, adult mosquitoes often adapt strategies to evade control through the development of behavioural and physiological resistance, such as reducing endophilic and endophagic behaviour, changes in diel biting rhythms [14], and developing insecticide avoidance [15].

Another set of mosquito population control techniques targets the aquatic stages. Aquatic life stages are generally more vulnerable to control measures, because they are restricted to the water body in which they hatch. The oldest, and still among the most efficient larval control methods is source reduction, a technique consisting of the eradication of mosquito larval breeding sites or steps taken to render them inaccessible, so that breeding is prevented [16]. Since full source control is unfeasible in many situations—most obviously in wetlands, irrigated rice cultivation, and regions with high precipitation rates—chemical insecticides, such as DDT, organophosphates, and methoprene [17], have been used as larvicides for mosquito control for many decades. Concerns over the negative ecological/environmental impacts attending the use of chemical insecticides have driven the search for environmentally safer alternatives, including *Bacillus thuringiensis israelensis* (*Bti*) and *Bacillus sphaericus* (*Bs*), entomopathogenic fungi, and natural enemies like nematodes, copepods, and fish [18–21]. Of these, *Bti* and *Bs* are among the most eco-friendly and efficient larvicides used to suppress mosquito populations and to reduce disease transmission, especially by *Culex* and *Anopheles* mosquitoes [2, 4, 18, 22–25]. However, the use of *Bacillus* larvicides is restricted by the economic constraints of application on wide spread ephemeral water bodies, their rapid inactivation through UV radiation [26–28] and sedimentation in the soil and leaf litter. This poor residual activity requires frequent reapplication for *Bti* and *Bs* larvicides [29–32], hampering intervention and efficacy [32–34]. Formulations that remain buoyant and do not sediment to the ground of water bodies could potentially increase the effectiveness of *Bti* and *Bs* at least by an order of magnitude as shown for *Anopheles* [35]. However, this possibility has been underresearched and underutilized, perhaps in part because of the lack of a suitable technique to achieve the required buoyancy [36].

In addition to increasing the longevity of *Bti* and *Bs* and the buoyancy of their carrier material, the potential of these control agents could be further enhanced through increasing the contact rate with the target organisms. By attracting mosquitoes to or arrestment of mosquitoes at the control agents, commonly called attract and kill, synergy in control can be expected [37–39]. If the attractant used is species specific, it will further improve the environmental friendliness of the intervention. For odorants to act in the desired manner, however, it is critical to deliver them at the most effective concentrations and ratios. Attractants can become unattractive or even repellent to the target insects if release rates are too high or when they are applied at improper ratios [40–44]. For several mosquito species, there is accumulating evidence that odorants play a significant role in the selection of oviposition sites by gravid females [45]. One example of such an odorant is the oviposition pheromone of *Cx*. *quinquefasciatus* (-)-(5*R*,6*S*)-6-acetoxy-5-hexadecanolide (henceforth abbreviated to AHD), which is released from droplets on the top of floating egg rafts, and which induces conspecific females to lay their eggs nearby [37, 46–51]. AHD is frequently referred to as “Mosquito Oviposition Pheromone,” or MOP, though this is inaccurate and can be misleading, since only species of the genus *Culex* are known to produce and use AHD in their communication system.

Application of AHD for purposes of vector mosquito control is limited by the same factors as *Bti*/*Bs* application: AHD rapidly precipitates, diffuses, or breaks down after application onto the water surface layer. Thus, the efficacy of both *Bti* and *Bs* control agents and the AHD attractant could be greatly improved by their incorporation into a buoyant, slow-release carrier material. Wax emulsion matrices [52, 53] offer such potential.

Focusing primarily on a vector of lymphatic filariasis, *Cx*. *quinquefasciatus*, our research investigated 1) if wax emulsions that contain control agents such as *Bti* and *Bs* induce mortality in a dose, larval stage and time-since-application dependent manner in mosquito larvae 2) if wax matrices incorporating the *Culex* oviposition pheromone (AHD) attract gravid females in a species-specific, dose and time-since-application dependent manner, and finally, 3) whether some synergistic effect can be observed when combining the killing agents (*Bti*/*Bs*) with the attractant (AHD) under both laboratory and field-resembling conditions.

## Methods

### Insect culture

For laboratory assays, *Cx*. *quinquefasciatus* Say 1823 (Thai strain; obtained from the London School of Hygiene and Tropical Medicine), *Aedes aegypti* (Linnaeus 1762; Rockefeller strain obtained via Wageningen University from Bayer AG Monheim), *Anopheles arabiensis* (Patton 1905; Dongola strain; obtained from the International Atomic Energy Agency, Vienna, Austria) and *Anopheles gambiae* (Giles 1902; Kisumu strain; a strain brought from Kenya Medical Research Institute and colonized at the National Institute for Medical Research, Amani Research Centre since early 1982) were maintained in a controlled environment (27±1°C, 65±5% relative humidity (RH), and at a 12 h:12 h light: dark cycle). Larvae were reared in distilled water-filled plastic trays (20 cm × 30 cm × 10 cm) in groups of <500 (*Culex*, *Aedes*) or <250 (*Anopheles*) per tray and fed on fish food once a day (SuperVit— 8 Mix—Tropical and Tetramin, one tea spoon tip per day and tray). For semi-field assays with *Cx*. *quinquefasciatus*, we used a strain established by the Tropical Pesticide Research Institute (TPRI), Tanzania. Adults were kept in cages (30 cm × 30 cm × 30 cm) with ad libitum access to a 12% sucrose solution. To enable reproduction, female mosquitoes were fed on sheep blood (Håtunalab, Bro, Sweden) via a membrane feeding system (Discovery labs, Accrington, UK) or, partially, on a human arm (DLP Schorkopf for *Anopheles*). The participation of humans in blood-feeding mosquitoes during routine colony maintenance was approved and monitored by the Central Ethical Review Board in Sweden. For semi-field assays in Tanzania, *Cx*. *quinquefasciatus* (TPRI strain, Tanzania) were blood fed on rabbits according to Standard Operating Procedures approved by the Medical Research Coordinating Committee of the National Institute for Medical Research (NIMR, Tanzania; research permit NIMR/HQ/R.8a/Vol.IX/1584). Rabbits were kept following European Community guidelines and standards (http://www.dantes.info/Tools&Methods/Othertools/Docs/86.609.EEC.pdf). Other semifield mosquito rearing conditions were the same as described for the lab assays above.

### SPLAT

For all experiments, we used the commercial matrix SPLAT^™^ (Specialized Pheromone and Lure Application Technology, ISCA Technologies, Riverside, CA, USA), which is modified from similar matrices used by Atterholt et al. [52, 53]. The waxes and oils used in SPLAT are biodegradable [54, 55] and therefore SPLAT is suitable for sustainable pest management. Of the different varieties of SPLAT available from its manufacturers, we used SPLATblank and SPLATbac. SPLATblank did not contain attractants or control agents, whereas SPLATbac contained 83.3g *B*. *thuringiensis* Berliner 1915 subsp. *israeliensis* de Barjac 1980 (serovar H14, henceforth *Bti)* and 83.3g *B*. *sphaericus* Meyer and Neide 1905 (serotype H5a5b, strain 2362, henceforth *Bs*) per kilogram of formulation.

SPLAT has a putty-like consistency and is usually applied directly onto substrates. However, we observed during the beginning of the present study that freshly made dollops applied directly on water partly dissolve and spread like a film across the water surface. To create cohesive, buoyant dollops that slowly release both attractants and control agents, we first dried the dollops for 5 days prior to application, so that they kept their shape and floated on the water surface (henceforth SPLAT pellets, see below).

#### SPLAT and SPLATbac with *Culex* oviposition pheromone, AHD

To quantify the efficacy of SPLAT as slow release tool of semiochemicals on water surfaces, we chose the *Culex* oviposition pheromone (-)-(5*R*,6*S*)-6-acetoxy-5-hexadecanolide [56, 57]. We included AHD into either SPLAT (SPLATahd) or SPLATbac (SPLATbacAHD). We compared the proportion of egg rafts laid by *Cx*. *quinquefasciatus* in test and control water. To test the specificity of AHD treated SPLAT on the egg-laying behaviour of other mosquito species, we also included laboratory tests with SPLATahd with *An*. *arabiensis*, *An*. *gambiae* and *Ae*. *aegypti*.

#### SPLAT pellets

All substances incorporated into SPLAT were mixed into the SPLAT emulsion prior the pellet production procedure. We allowed SPLAT dollops to dry on aluminium foil for 5 days at 18–25°C, 40 to 60% RH. We used gloves throughout the study. Each pellet weighed on average 20 mg. SPLATahd pellets contained approximately 8 μg of (-)-(5*R*,6*S*)-6-acetoxy-5-hexadecanolide (Bedoukian Research Inc., USA).

#### SPLATbac and mortality assays

The mortality of mosquito larvae following exposure to SPLATbac was evaluated in laboratory assays. Pilot studies showed that undiluted SPLATbac, even as small pellets (in the range of 1 mg), caused 100% larval mortality in our standard experimental trays. Therefore, four serial dilutions of SPLATbac were tested by mixing it with SPLATblank (10^−1^–10^−4^). One pellet weighing 20 mg was added to each larval rearing tray (20 cm × 30 cm × 10 cm) containing 1 L of distilled water. Controls with SPLATblank pellets accompanied the tests with SPLATbac. SPLATbac-induced mortality was tested in two experiments. In the first experiment, we added two egg rafts (~160 *Cx*. *quinquefasciatus* eggs) to each larval rearing tray, whereas in the second experiment, we added thirty 3^rd^ instar larvae. Feeding and rearing was continued as described above. Each treatment was replicated at least four times. Larval mortality was scored at 6, 12, and 24 h post treatment, followed by daily observations (every 24 h) until adult emergence.

To test the larvicidal persistence of SPLATbacAHD, we scored mortality of larvae at day 1, 5, 10, 20, and 40 after application of SPLATbac (10^−1^) and the SPLATblank by transferring thirty 3^rd^ instar larvae, or two egg rafts (~160 eggs each), to each of these trays. Otherwise, the methods were as in the mortality assays mentioned above.

### Laboratory oviposition choice assays

Blood-fed females in their first gonotrophic cycle (6 to 24 hours post blood meal) were transferred to 30 cm × 30 cm × 30 cm cages (BugDorm, Megaview, Taiwan) more than 24 h prior the start of the experiments with *ad libitum* access to a 12% sucrose solution placed in the centre. Single or multiple females of either *Cx*. *quinquefasciatus*, *Ae aegypti*, *An*. *arabiensis* or *An*. *gambiae* (as specified) were given a dual choice between two 250 mL disposable cups each containing 50 mL distilled water, containing either SPLATblank or SPLATahd. Disposable cups were conventional paper or plastic cups, which we only used once and then discarded. For each set of oviposition choice experiments we used the same type and brand of 250 mL cups. Oviposition experiments with *Aedes* included a filter paper disc (diameter 9 cm) on the inner wall of each oviposition cup, as *Aedes’* prefers to deposit eggs on moist edges of water bodies rather than directly onto the water. The position of cups was alternated between experiments to minimize positional bias. Test conditions were the same as rearing conditions. Eggs were counted after each experimental night. The longevity of SPLATahd in inducing oviposition in *Cx*. *quinquefasciatus* was tested by using dollops 1, 5, 10, 20, and 40 days after application in water. Methods otherwise followed those mentioned above.

### Oviposition choice assays in field condition emulating screen spheres

Experiments were carried out in screened greenhouses (so-called mosquito spheres) in the field located at the National Institute for Medical Research, Amani Research Centre (0510'220"S, 3846'733"E), in Muheza, Tanzania [58]. The mosquito spheres (12.2 m long, 8.2 m wide, and approximately 5 m high) contained a traditional mud hut and natural vegetation, including different grass, flower, and shrub species and three small trees no taller than 2.4 m. To better mimic the appearance of natural oviposition sites, we used locally made clay bowls (~ 0.2 m in diameter and 0.09 m high) and positioned them so that the top bowl margins were level with the surrounding ground. We filled the bowls with soil and water from a nearby natural *Culex* oviposition site. The positions of the 32 bowls within the sphere were approximately equidistant from each other. We applied either SPLATbacAHD (treatment) or SPLATblank (control) in the morning preceding the experiment. Treatments and controls were alternated within and between experiments to minimize positional bias. A cage containing between 150 to 300 gravid *Cx*. *quinquefasciatus* was placed in the hut (“indoors”) and opened in the early evening so that the females could leave the cage. In the morning after the experiment, each clay bowl was checked for egg rafts and the number noted. Because the number of total egg rafts laid in each of the six experimental nights differed, we used the proportion of egg rafts laid in treatment vs. control for comparison rather than the total number of egg rafts in each bowl. Results were discarded when rainfall led to overflowing of water in any of the clay bowls. After counting, egg rafts collected from control (SPLAT) and test formulations (SPLATbac or SPLATbacAHD) bowls were transferred to two separate 5 L plastic buckets, respectively. Only one SPLAT or SPLATbac pellet was allowed to remain per L of above mentioned oviposition site material in those buckets. The number of emerging larvae from the sphere assays was estimated in decadic steps (nearest to 10, 20, 30 etc…) and mortality was observed for a period of 72 h post emergence.

### Statistical analysis

For oviposition choice results, the non-normally distributed percentages of total number of eggs or egg rafts generally required non-parametric statistical approaches, especially when data did not meet the assumptions of equal variance (Spearman rank correlation p>0.05) and normal distribution (Shapiro-Wilk test p>0.05). For comparison of proportions within the same experiment, we used Wilcoxon signed rank tests, and Mann-Whitney U tests between two separate oviposition experiments. When multiple data sets were compared, we used Kruskal-Wallis ANOVAs, followed by Dunn’s pairwise comparison after finding significant differences, unless assumptions for parametric ANOVA were met. For cases meeting the latter criteria, we calculated conventional ANOVAs, followed by Tukey’s pairwise comparisons. For pairwise comparisons in longevity studies of AHD, we chose the less conservative post-hoc Fishers’ LSD. This was chosen under the assumption of a decline of pheromone release over time (Atterholt et al. 1998, 1999) and following pilot studies strongly indicating a gradual decrease of oviposition attraction over time. Box plots in this paper show the range from 1^st^ to 3^rd^ quartile with median (bold line) and arithmetic mean (dotted line) plus 5/95% percentiles (whiskers). As statistical software package, we used Sigmastat 4.0 (Systat Software Inc.).

## Results

### Larvicidal action of SPLATbac (formulations containing *Bti* + *Bs*)

In the laboratory, SPLATbac caused 100% mortality of *Cx*. *quinquefasciatus* larvae within 48 h after application of undiluted SPLATbac in all tested amounts (20 mg L^-1^, 8 mg L^-1^, 1 mg L^-1^ per litre), irrespective of larval instar (1^st^ instar: N = 6 x 60 larvae; or 3^rd^ instar: N = 5 x 30 larvae). As 100% mortality does not permit the detection of efficiency and longevity thresholds of SPLATbac, we henceforth used dilutions of SPLATbac (10^−1^ to 10^−4^).

**Fig 1:**
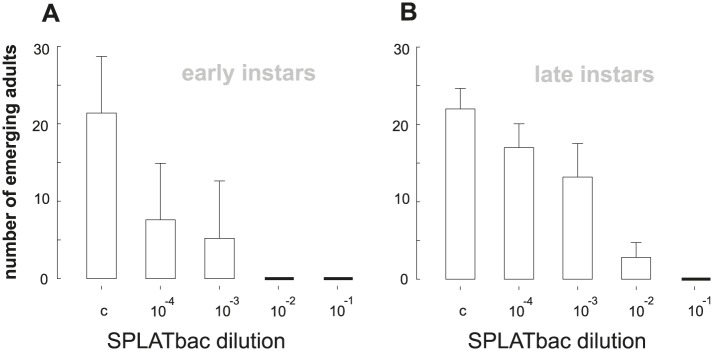
Adult emergence from A) early instars and B) late instars (Cx. quinquefasciatus) added to different SPLATbac dilutions (control, c = SPLATblank). The arithmetic mean plus SD are shown for each concentration.

SPLATbac significantly reduced the developmental success from egg or 3^rd^ larval instar to adult in a concentration dependent manner (Fig 1; Kruskal-Wallis ANOVA on Ranks; hatching eggs: H_df = 4_ = 17.55, p = 0.002; N_[each concentration]_ = 5; late instar: H_df = 4_ = 21.489, p < 0.001; N_[each concentration]_ = 5). Compared to the control (SPLATblank), we observed significantly elevated mortality rates following exposure to SPLATbac treatments in recently emerged 1^st^ instar larvae (all SPLATbac dilutions) and 3^rd^ instar larvae (SPLATbac dilutions down to 10^−3^). SPLATbac induced 100% mortality in all larvae before they reached adulthood at dilutions of 10^0^−10^−2^ for 1^st^ instar, and at dilutions of 10^0^−10^−1^ in 3^rd^ instar larvae.

#### SPLATbac longevity

**Fig 2:**
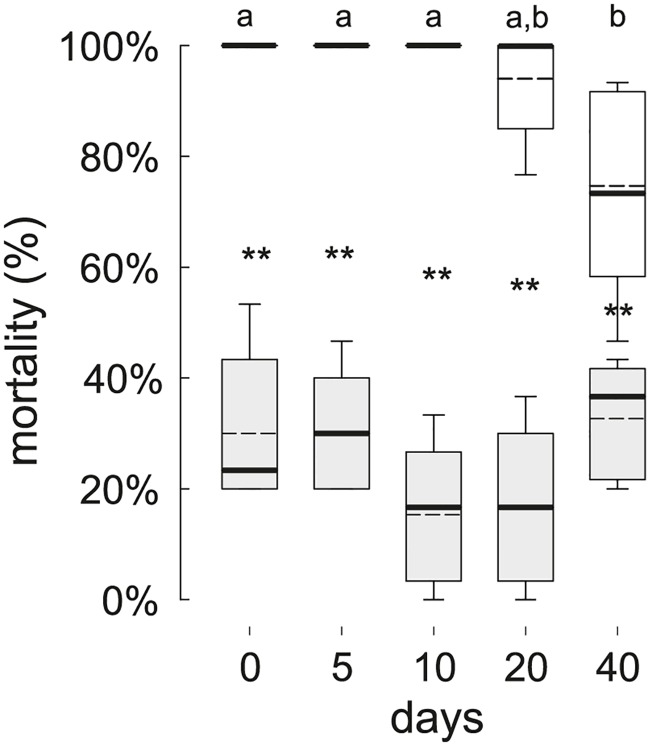
Mortality of 3^rd^ instar *Cx*. *quinquefasciatus* after transfer to water bodies with different post-SPLAT application periods. Grey box plots indicate the mortality values obtained for the control (SPLATblank), while the white box plots represent those for the treatment (SPLATbac 10^−1^). Different letters indicate significant differences (Dunn’s pairwise multiple comparisons, p < 0.05) between SPLATbac post-application periods. Asterisks indicate significant differences between SPLATbac and SPLATblank treatments from the same post-application day (** = p < 0.01).

**Fig 3:**
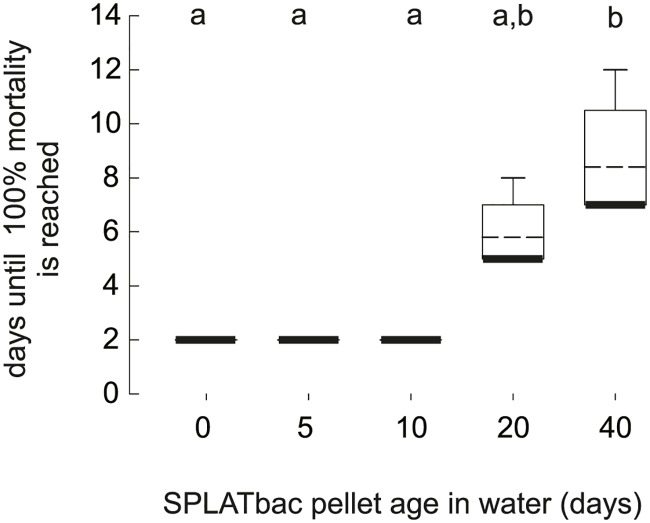
Aging of SPLATbac pellets slows mortality of newly hatched *Cx*. *quinquefasciatus* larvae. Box plots are shown with medians (bold lines) and arithmetic means (dashed lines). Different letters indicate statistically significant differences (Dunn’s pairwise multiple comparisons, p < 0.05) between SPLATbac post-application periods.

After application into water, SPLAT pellets remained on the water surface until the end of our experiments. SPLATbac pellets (10^−1^) caused significant mortality (Mann-Whitney rank sum tests, p < 0.05, Fig 2) until 40 days after application. The survival rate of 3^rd^ instar larvae, however, increased in SPLATbac treated water after 20 or more days (Fig 2, Kruskal-Wallis ANOVA on Ranks, N_[each concentration]_ = 5; among SPLATbac treatments: H_df = 4_ = 18.85, p < 0.001; among control SPLATblank treatments: H_df = 4_ = 7.4, p = 0.116). No neonate 1^st^ instar larvae (N_[each concentration]_ = 5 x 2 egg rafts) survived to adulthood even in 40-day-old SPLATbac (10^−1^) treated water, although the time to death of 1^st^ instar larvae increased in 20 days and older waters (Fig 3; Kruskal-Wallis ANOVA on Ranks, H_df = 4_ = 23, p < 0.001, N_[each concentration]_ = 5).

### The effect on egg-laying of *Culex* oviposition pheromone (AHD)-containing formulations

#### SPLATblank (untreated SPLAT)

*Cx*. *quinquefasciatus* preferred to oviposit in SPLATblank treated water cups over cups with distilled water only (median = 60%, Q1 = 57.1%, Q3 = 77.8%; mean difference to paired control = 28.3% ±21.9SD; Wilcoxon signed rank W_N = 7_ = 21, P = 0.031, r = 0.59; N = 7 x 14 females, 65 egg rafts in total). However, this effect vanished when covering the visual cues with filter paper (mean difference to paired control = 1.9% ±33.1SD; W_N = 7_ = 0, P > 0.05, median = 53.3%, Q1 = 37.5%, Q3 = 66.7%; N = 7 x 14 females, 64 egg rafts in total). Thus, care was taken to provide identical visual cues for subsequent bioassays in which we compared the effect of any SPLAT pellet formulation against a control.

#### SPLATahd (SPLAT containing acetoxy hexadecanolide)

*Culex*. Single *Cx*. *quinquefasciatus* preferred to oviposit in water cups containing a SPLATahd pellet to those containing a SPLATblank pellet (mean difference to paired control = 61.5% ±80.4SD; Wilcoxon signed rank W_N = 24 pairs_ = 216, p = 0.002, median = 100%, Q1 and Q3 = 100%, r = 0.52). This was significantly different (mean difference = 28.8%, Mann-Whitney U = 231.5; p = 0.032, r = 0.25; N_SPLATahd_ = 26, N_SPLATblank_ = 25) from the response observed in choice tests with cups containing SPLATblank pellets only (mean difference to paired control = 4% ±102SD; W_N = 25 pairs_ = 13, p > 0.05, median = 0%, Q1 = 0%, Q3 = 100%). Groups of *Cx*. *quinquefasciatus* (N = 8 x 14 females, 99 egg rafts in total) also preferred to oviposit in SPLATahd treated water cups compared to SPLATblank (mean difference to paired control = 80.8% ±25.1SD; W_N = 8 pairs_ = 36, p = 0.008, median = 96.7%, Q1 = 80.8%, Q3 = 100%, r = 0.64).

*Aedes*. *Ae*. *aegypti* females tested singly (one female per cage) did not prefer SPLATahd over SPLATblank (Wilcoxon signed rank W_N = 10 pairs_ = 13, p > 0.05, n = 359 eggs). There was no difference to control experiments (Mann-Whitney U = 42.5; p > 0.05; N_SPLATahd_ = 10, N_SPLATblank_ = 10) where both waters were treated with SPLATblank (W_N = 10 pairs_ = 1, p > 0.05). Similar results were obtained for cohorts of seven *Ae*. *aegypti* individuals: Single *Ae*. *aegypti* (seven females per cage) did not lay significantly different egg numbers when SPLATblank was compared with the control (identical SPLATblank; W_N = 7 pairs_ = 6, p > 0.05, n = 1447 eggs) or when compared with SPLATahd (W_N = 6 pairs_ = 11, p > 0.05, n = 1312 eggs). There was no significant difference between both treatments (U = 19; p > 0.05; N_SPLATahd_ = 6, N_SPLATblank_ = 7).

*Anopheles*. No difference in oviposition preference was observed between SPLATahd and the controls for either of both tested *Anopheles* species.

*An*. *arabiensis*. Single *An*. *arabiensis* (one female per cage) did not lay significantly different egg numbers when SPLATblank was compared with SPLATblank (W_N = 7 pairs_ = 1, p > 0.05, n = 377 eggs) or when compared with SPLATahd (W_N = 7 pairs_ = 8, p > 0.05, n = 530 eggs). There was no significant difference between both treatments (U = 21; p > 0.05; N_SPLATahd_ = 7, N_SPLATblank_ = 7). There was also no significant difference between treatments when groups of seven females per cage were tested (U = 12; p > 0.05; N_SPLATahd_ = 6, N_SPLATblank_ = 6). Groups of *An*. *arabiensis* did also not lay significantly different egg numbers when SPLATblank was compared with SPLATblank (W_N = 5 pairs_ = 9, p > 0.05, n = 1323 eggs) or when compared with SPLATahd (W_N = 5 pairs_ = 1, p > 0.05, n = 1622eggs).

*An*. *gambiae*. Single *An*. *gambiae* did not lay significantly different egg numbers when SPLATblank was compared with SPLATblank (W_N = 7 pairs_ = 1, p > 0.05, n = 338 eggs) or when compared with SPLATahd (W_N = 6 pairs_ = 3, p > 0.05, n = 530 eggs). There was no difference between both treatments (U = 15; p > 0.05; N_SPLATahd_ = 7, N_SPLATblank_ = 7). There was also no significant difference between treatments when groups of seven females per cage were tested (U = 19; p > 0.05; N_SPLATahd_ = 6, N_SPLATblank_ = 7). Groups of *An*. *gambiae* did not lay significantly different numbers of eggs when SPLATblank was compared with SPLATblank (W_N = 7 pairs_ = 6, p > 0.05, n = 2844 eggs) or when compared with SPLATahd (W_N = 6 pairs_ = 3, p > 0.05, n = 892 eggs).

#### *Bti* and *Bs* contained in SPLATbac did not affect the oviposition choice of *Cx*. *quinquefasciatus*

**Fig 4:**
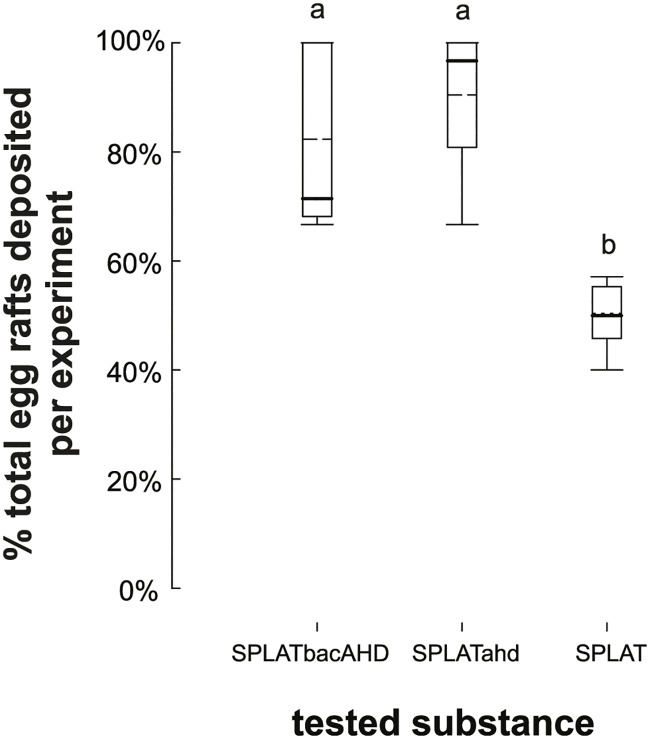
Proportion of *Cx*. *quinquefasciatus* egg rafts laid in treatment (SPLATbacAHD, SPLATahd) compared to control (SPLATblank). Box plots with both medians (bold lines) and means (dotted lines) are shown. Same letters indicate that treatments did not differ significantly (p < 0.05) from each other (Tukey pairwise comparisons after ANOVA).

*Bti/Bs* containing pellets with AHD were equally effective at killing larvae whether or not AHD was included (N = 7 x 14 females; 65 egg rafts; mean difference of SPLATbacAHD to paired control SPLATbac = 65% ±32.9SD, Wilcoxon signed rank W_N = 7 pairs_ = 28, p = 0.016, median = 71.4%, Q1 = 69.2%, Q3 = 100%, r = 0.76). SPLATahd and SPLATbacAHD were preferred by gravid *Culex* females over SPLATblank (Fig 4; ANOVA F_df = 2, 22_ = 24.310, p < 0.001, ω^2^ = 0.67; mean difference_[SPLATblank vs SPLATahd]_ = 40.2%; Tukey q = 9.354, p <0.001; mean difference_[SPLATblank vs SPLATbacAHD]_ = 32.3%; Tukey q = 7.252, p < 0.001), while females did not differentiate among them (mean difference = 7.9%, Tukey q = 1.785, p = 0.432). Hence, no decrease was observed in *Cx*. *quinquefasciatus* oviposition preference when control agents were included (SPLATbacAHD vs. SPLATbac). Mortality was 100% in SPLATbac and SPLATbacAHD both for neonate 1^st^ instar (N = 6 x 2 egg rafts) and 3^rd^ instar larvae (N = 6 x 30) (SPLATbac/SPLATblank ratio 1:10). We thus also conclude that the inclusion of AHD does not change the toxicity of SPLATbac.

#### SPLATbacAHD remains attractive for more than two weeks

**Fig 5:**
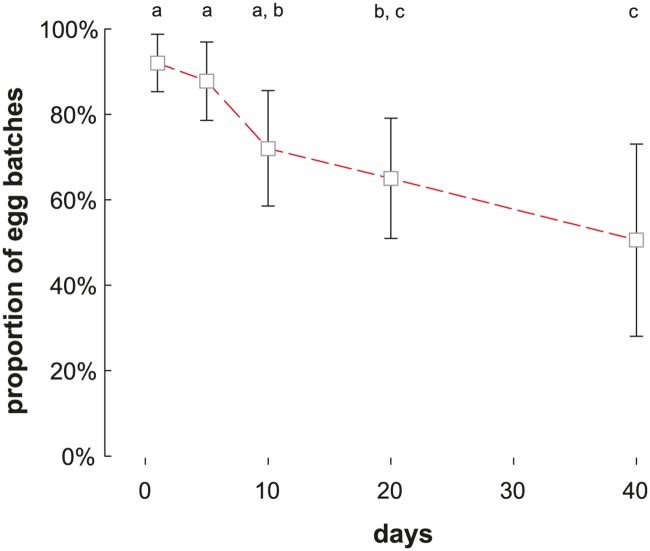
Proportion (±2SE) of *Cx*. *quinquefasciatus* egg rafts oviposited in SPLATbacAHD treated water over time after application. Means sharing the same letter indicate no significant differences (Fisher LSD multiple comparisons, p > 0.05).

The preference of *Cx*. *quinquefasciatus* to lay eggs in SPLATbacAHD treated water decreased with time after application (Fig 5, (N_[per longevity measure point]_ = 9 x 14 females) 320 egg rafts; ANOVA F_df = 4, 44_ = 5.640, p = 0.001, ω^2^ = 0.29; Fig 5). There was no change in preference to the controls (149 egg rafts; ANOVA F_df = 4, 44_ = 0.336, p = 0.852). Water with SPLATbacAHD pellets remained significantly more attractive until the 20^th^ day following application (N_[per treatment]_ = 9 x 14 females; Mann-Whitney U, p < 0.05) than control pellets (SPLATbac without AHD). By the 40^th^ day post-application SPLATahd pellets had noticeably disintegrated, and fungi were frequently observed growing on the pellets. All larvae that hatched from eggs laid in either SPLATbac or SPLATbacAHD during the above-mentioned tests died (see also paragraph on SPLATbac longevity above), irrespective of pellet age.

### SPLATbacAHD under field emulating conditions

**Fig 6:**
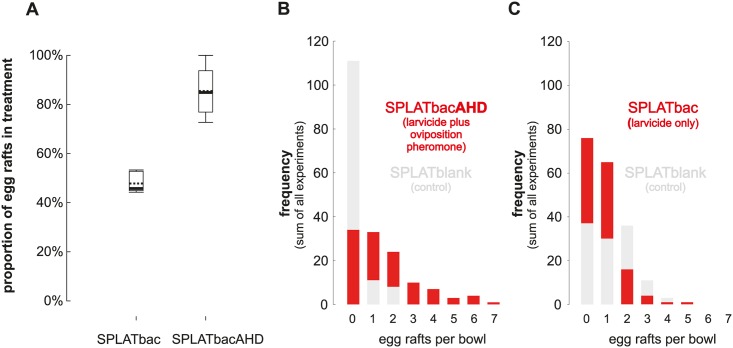
**Proportion of Cx. quinquefasciatus egg rafts laid in mosquito spheres either receiving SPLATbac larvicidal treatment (A) with or without the oviposition pheromone AHD in half of the 32 oviposition bowls containing natural oviposition site material.** The remaining half of the bowls in each treatment contained SPLATblank as a control. (B, C). The frequency distribution of the number of egg rafts per bowl in mosquito spheres receiving either SPLATbacAHD treatment (red, B) or SPLATbac treatment (red, C) as compared to the frequency distribution of the controls (SPLATblank; grey in both B and C) (N = 6 different days x 150 to 300 gravid females).

Under field resembling conditions (mosquito spheres) the relative proportion of *Cx*. *quinquefasciatus* eggs laid in SPLATbacAHD containing bowls (16 of 32) compared to control bowls (remaining 16 bowls containing SPLATblank) was comparable to that observed under laboratory conditions (Fig 6A). A significantly higher proportion of egg rafts (mean difference = 37.6%, Mann-Whitney U = 0; p = 0.002, r = 0.83; N_SPLATbacAHD_ = 6, N_SPLATbac_ = 6) compared to the control (SPLATblank containing bowls) was laid in the SPLATbacAHD containing bowls (mean difference to paired control = 70.9% ±19.5SD; Wilcoxon signed rank W_N = 6 pairs_ = 21, p = 0.031, median = 85.1%, Q1 = 76.9%, Q3 = 93.8%; r = 0.64; 1100 females and 185 egg rafts in total) than in SPLATbac containing bowls (mean difference to paired control = 4.3% ±8.1SD; Wilcoxon signed rank W_N = 6 pairs_ = 15, p = 0.156, median = 45.8%, Q1 = 44.9%, Q3 = 52.8%; 1150 females and 187 egg rafts in total). The number of egg rafts found in each clay bowl (32 bowls per experiment) ranged from zero to seven (Fig 6B and 6C). Between trials, clay bowls were shifted between treatment and control. Across all bowls, the average number of egg rafts did not differ between experiments (Kruskal-Wallis H_df = 31_ = 24.435, p = 0.792). Similarly, odd and even numbered bowls did not differ in the average number of egg rafts (difference between odd and even numbered clay bowls = 0.572%; t-test t_df = 30_ = 0.88, p = 0.381). Positional bias therefore did not appear to account for the “clumped” raft distribution (Fig 6B).

#### Intervention with SPLATbac and SPLATbacAHD in mosquito spheres

**Fig 7:**
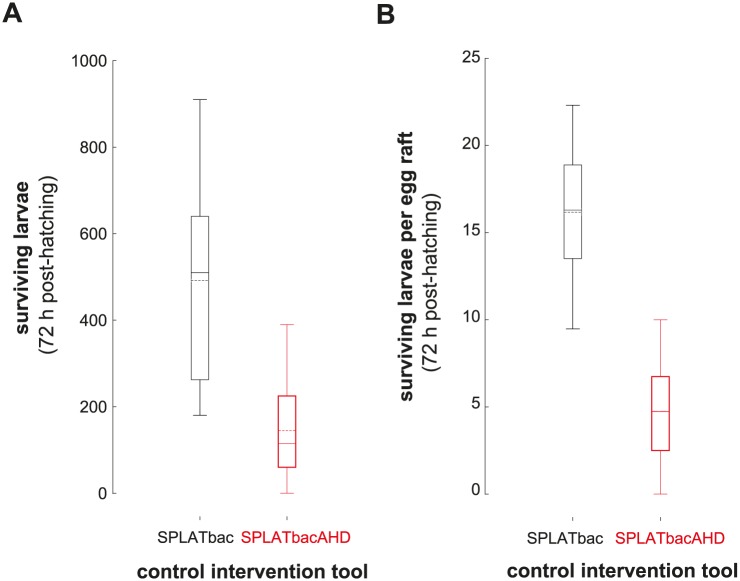
Number of surviving *Cx*. *quinquefasciatus* larvae 72 h-post hatching in the mosquito sphere. **(A)** Uncorrected and **(B)** corrected (divided by the total egg raft number laid per experiment).

All larvae hatching from those egg rafts laid into SPLATbac or SPLATbacAHD treated water bodies died within 72 hours under field emulating conditions. However, significantly fewer larvae survived per experiment in mosquito spheres receiving SPLATbacAHD treatment compared to spheres with SPLATbac treatment (Fig 7A; mean difference = 346 larvae, Mann-Whitney U = 2; p = 0.009, r = 0.74, N_SPLATbacAHD_ = 6, N_SPLATbac_ = 6). This also held true when correcting the numbers due to the different total number of egg rafts laid per experimental night (Fig 7B; mean difference = 11.5 larvae per egg rafts and night, Mann-Whitney U = 1; p = 0.004, r = 0.79, N_SPLATbacAHD_ = 6, N_SPLATbac_ = 6). The incorporation of AHD thus appeared to enhance larval mortality within the mosquito spheres beyond what was achieved by SPLATbac with only *Bti/Bs*.

## Discussion

Larvicidal tools, particularly those with minimal risks to the environment and non-target species, can be an efficient instrument in suppressing mosquito populations to combat the transmission of mosquito-borne diseases, such as lymphatic filariasis and malaria in the tropics and elsewhere. Thus, much research has focused on improving larvicidal efficacy, costs, acceptability, and impact on both environment and human health [2, 4, 18, 22–25, 31, 34, 36, 59–65]. The wax matrix evaluated in the current study could contribute significantly to the development of longer lasting, efficient, cost-effective, and sustainable mosquito larviciding tools. Such tools are much sought after by authorities working on the intervention of lymphatic filariasis and other mosquito transmitted diseases [3, 4, 13, 21, 32, 66–71]. Our results provide “proof of principle” that wax emulsion matrices that combine control agents and attractants can synergistically increase these tools’ impact on vector mosquito populations. Incorporating oviposition attractants in wax matrices such as SPLAT increases the likelihood of contact between larvae and the control agent, while the slow release of control agent by SPLAT greatly extends the duration over which the application can be effective.

### Efficacy of SPLATbac in inducing mortality

Our results demonstrate that SPLATbac slowly released *Bti* and *Bs* into the water in proportions sufficient to kill-off aquatic life stages of mosquitoes, at least under the small water body conditions evaluated in these studies. The varieties of *Bti* and *Bs* used in the present study were as effective in combination as described in previous studies (e.g. [29, 62, 72, 73]). A combination of *Bti* and *Bs* not only substantially improves larvicidal efficacy against *Culex*, but also suppresses the selection process that results in the building up of larvicide resistance [74, 75]. Our study determined that 17 μg per SPLAT pellet (20 mg) for each larvicide (*Bti/Bs*) to be sufficient to kill off all early and late instar larvae (1^st^ instar and 3^rd^ instar) prior reaching adulthood in one litre of water. We did not determine the release rates of *Bti* and *Bs* into water by the different SPLATbac formulations, but the biological longevity demonstrates that the release is relatively slow. It should be noted that the longevity of the formulation was assessed only under laboratory conditions. Considering that stability of SPLAT is well established under field conditions for numerous other semiochemicals and active ingredients [76–79], we anticipate that SPLATbac longevity would be similarly prolonged in the field. It is well known that young mosquito larvae are much more susceptible to *Bti* and *Bs* than later instars [36]. In our study, the differential susceptibility may have been further augmented by the longer exposure period of 1^st^ instar larvae compared to 3^rd^ instar larvae.

#### Longevity and buoyancy of SPLATbac

Increasing the longevity of formulations reduces the need for frequent reapplication and reduces costs [31, 63, 68, 71]. A more diluted SPLATbac at a 10^−1^ dilution of SPLATbac reliably killed off all larval stages until 10 days after application and only lost about 10% of its efficacy by the 20^th^ post-application day. Even at the 40^th^ post-application day, mortality was 100% for first instar larvae, whereas only some residual toxicity of SPLATbac was found for late instars (3^rd^ instar larvae), illustrating the significantly higher susceptibility of early instars to *Bti* and *Bs*. Further field experiments are needed to evaluate to what extent the improved longevity reported here also holds under natural conditions and can be used to reduce intervention frequencies (see e.g. [31, 36]). Another advantage of the SPLAT matrix formulation is its buoyancy, which extended over the whole test period of 40 days. This prevents sedimentation and sustains release of *Bti* and *Bs* within the top layers of water bodies where mosquito larvae occur most frequently [80]. The buoyancy of SPLATbac is also critical for the release of semiochemicals that attract adult mosquitoes, thereby synergizing vector control using *Bti* and *Bs* (see below).

### Oviposition attraction using wax emulsions

Our results demonstrate that mosquito oviposition attractants in wax emulsions can modulate the behaviour of gravid females and be used in surveillance and control. By exploiting the chemosensory system of the target species e.g. by utilizing species-specific attractants or pheromones, the sensitivity of surveillance and the efficacy of control can be greatly enhanced. Here we show that gravid *Cx*. *quinquefasciatus* consistently preferred bodies of water treated with SPLAT containing small amounts (0.4 μg/mg) of *Culex* oviposition pheromone (AHD), irrespective of the presence of *Bti* and *Bs*. Since the presence of AHD did not reduce SPLATbac induced mortality, our results show that attractants and control agents can be successfully combined into a single formulation without negatively impacting the efficacy of either component. The buoyancy of our SPLAT pellets facilitates the emission of volatiles from the wax matrix into the air, which otherwise would risk entrapment in water, depending on the properties (e.g. polarity, vapour pressure). Application on a floating structure appears critical for AHD to be able to attract mosquitoes [39, 48, 51, 81–86].

None of the other species tested (*Ae*. *aegypti*, *An*. *gambiae*, *An*. *arabiensis*) preferred to oviposit into AHD treated formulations. This is similar to findings of Hwang et al. [82] on *An*. *quadrimaculatus* and *Ae*. *aegypti*. Although attraction of multiple vector species would have been advantageous, it is encouraging to note that the opposite effect—a repellent or deterrent effect of AHD in *Aedes* or *Anopheles*—was not observed, either. Interspecific interactions, such as reported in *An*. *gambiae* [87] are clearly not mediated by AHD. It is therefore advantageous that AHD does not deter other important vector species from ovipositing. Further studies should investigate whether other mosquito oviposition attractants for *Culex* or other mosquito genera influence attraction of AHD and vice versa.

#### Longevity of SPLATahd-mediated attraction

Some AHD-induced oviposition attraction was observed at least until the 20^th^ day post-application. Since many of the pellets started to show obvious signs of degradation, presumably due to activity of microorganisms around that time, the decrease in attraction was likely caused in part by biodegradation and not merely by the depletion of pheromone from the pellets into the water bodies. Similarly, the decrease in mortality with time was likely in part due to disintegration and microbial breakdown. The biodegradability, partly a function of the formulation of SPLAT, is a desirable quality in terms of environmental friendliness. Depending on the viscosity of the SPLAT formulation, field studies on SPLAT volatile emission above ground [78, 88] show pheromones to be released in sufficient amounts to mediate male moth behaviour for up to 12 weeks after application. Future research could investigate whether compounds that slow microbial growth can be used to increase the longevity of SPLAT even further. We infer that the current formulation of SPLATbacAHD pellets may remain attractive for more than 2 weeks after application into water bodies.

### Synergistic effects by combination of attractant and control agent under field emulating conditions

Our semi-field experiments demonstrated that AHD synergized mortality caused by *Bti* and *Bs*. Because female mosquitoes preferred to oviposit in bowls with AHD the overall larval survival rate within these spheres was reduced to one third compared to spheres where we applied SPLATbac without AHD (Fig 7). This synergistic effect by SPLATbacAHD needs further evaluation under full field/natural conditions [31, 36, 63, 68, 71, 85]. A thorough evaluation should take into account factors that could override the preference for larval breeding sites containing AHD (e.g. presence of food, predators or competitors; [45, 89–92]), as well as factors that directly affect the attraction to AHD, such as the range of attraction by AHD (not assessed here), and the *Culex* population density (density of other AHD-emitting egg rafts). Such factors will determine to what extent the addition of AHD can increase the cost-effectiveness of SPLATbac.

#### Advantages of wax emulsions

Wax emulsions such as SPLAT possess several technical and environmental advantages. They are seen as environmentally friendly since they are composed of biodegradable components. Also, wax emulsion formulations can be adapted regarding floatability, stickiness, dollop or pellet portions, control or behaviour mediating agent concentrations and biodegradability times [52, 53]. Wax emulsions can be applied manually or by machines, on the ground or from airborne vehicles. The SPLAT formulations tested here have a good chance to fulfil important requirements [3, 13, 31, 36, 70], including the suitability for long distance transport, storability under tropical climate conditions, easy application, non-hazardousness and environmental friendliness, high efficacy against mosquito vectors and cost-effectiveness. SPLAT can readily be used as carrier of other larvicidal agents if necessary for reasons of i) vector species and population specificity, ii) control efficacy and iii) resistance issues [93]. The latter was reported sporadically for *Bti* and *Bs* [36, 74, 75, 94–96].

Because of its flexibility, ease of application, and biodegradability, SPLAT has already been used successfully against several crop pest species, either utilizing mating disruption (sex pheromone embedded SPLAT; e.g. [79, 97, 98] or attract and kill techniques (sex pheromone plus insecticides embedded SPLAT formulations; [99, 100]). It is likely that hardened wax emulsions pellets, such as those used in our study, could also work if applied ‘prophylactically’ prior to rainfall. One batch of SPLATbacAHD that we left within the sphere for five days in the sun after an aborted experiment did not fail in attracting gravid *Cx*. *quinquefasciatus* laying eggs and caused 100% larval mortality. Probably, the dried upper layers of the wax emulsion pellets act as an UV-barrier protecting the content within. However, these claims would need to be verified under field conditions in future studies.

#### Potential of SPLATbac: caution and future research

This study clearly demonstrates the potential of SPLATbac in vector control. However, a thorough analysis of environmental effects of SPLATbac for aquatic environments and non-target aquatic organisms has not been done. Given the sensitivity of aquatic ecosystems this needs to be given due research attention. Especially *because* the two larvicides *Bti* and *Bs* are widely claimed to be environmentally safe and “harmless” to non-target organisms [18, 36], future field studies must seek to continuously provide evidence regarding potential direct *and* indirect negative side effects of SPLATbacAHD under field conditions [101]. While suppressing mosquito populations by SPLATbac treatments in man-made and ecologically less valuable structures such as borrow pits and sewage canals are probably of less environmental concern, the application of SPLATbac in more fragile fresh water ecosystems and drinking water reservoirs needs to be carefully analysed in relation to e.g. effects on natural enemies, beneficial organisms, plant and animal health, and drinking water quality.

Our study shows that wax emulsions are well suited to the combination of mosquito attractants and control agents. We hope that the presented tools will improve the cost effectiveness and environmental sustainability of future vector control programs [1, 12, 69]. The flexibility, buoyancy, and slow release of control agent, along with the synergy achieved by the addition of oviposition attractants appear advantageous over current control techniques. Intervention programs targeting mosquito vectors other than *Cx*. *quinquefasciatus* [1, 3, 4, 36] could embed attractants in SPLAT that target these species, such as those recently identified for *An*. *gambiae* and *Ae*. *aegypti* [45, 102–104]. As important tropical vector-borne diseases disproportionally affect developing countries [105–107], cheaper and technologically less demanding larvicidal tools could substantially improve mosquito control interventions where they are most needed. Further research should include field trials and calculations of cost-effectiveness to evaluate the actual sustainability of the presented tools in the real world.

## References

[1] WHO. World malaria report 2014. Geneva: World Health Organization; 2014.

[2] WHO. Dengue: guidelines for diagnosis, treatment, prevention and control: World Health Organization; 2009. 23762963

[3] WHO. Global strategy for dengue prevention and control 2012–2020: World Health Organization; 2012.

[4] WHO. Progress report 2000–2009 and strategic plan 2010–2020 of the global programme to eliminate lymphatic filariasis: halfway towards eliminating lymphatic filariasis. Geneva; 2010.

[5] Chandy A, Thakur AS, Singh MP, Manigauha A. A review of neglected tropical diseases: filariasis. Asian Pac J Trop Med. 2011;4(7):581–6. 10.1016/S1995-7645(11)60150-8 . 21803313

[6] Curtis C. Should DDT continue to be recommended for malaria vector control? Medical and veterinary entomology. 1994;8(2):107–12. 8025316 10.1111/j.1365-2915.1994.tb00147.x

[7] Roberts DR, Manguin S, Mouchet J. DDT house spraying and re-emerging malaria. Lancet. 2000;356(9226):330–2. 10.1016/s0140-6736(00)02516-2 . 11071203

[8] Esu E, Lenhart A, Smith L, Horstick O. Effectiveness of peridomestic space spraying with insecticide on dengue transmission; systematic review. Trop Med Int Health. 2010;15(5):619–31. 10.1111/j.1365-3156.2010.02489.x . 20214764

[9] N'Guessan R, Asidi A, Boko P, Odjo A, Akogbeto M, Pigeon O, et al An experimental hut evaluation of PermaNet((R)) 3.0, a deltamethrin-piperonyl butoxide combination net, against pyrethroid-resistant *Anopheles gambiae* and *Culex quinquefasciatus* mosquitoes in southern Benin. Trans R Soc Trop Med Hyg. 2010;104(12):758–65. 10.1016/j.trstmh.2010.08.008 . 20956008

[10] Malima R, Tungu PK, Mwingira V, Maxwell C, Magesa SM, Kaur H, et al Evaluation of the long-lasting insecticidal net Interceptor LN: laboratory and experimental hut studies against anopheline and culicine mosquitoes in northeastern Tanzania. Parasit Vectors. 2013;6(1):296 10.1186/1756-3305-6-296 24499488 PMC4028879

[11] van den Berg H, Kelly-Hope LA, Lindsay SW. Malaria and lymphatic filariasis: the case for integrated vector management. Lancet Infect Dis. 2013;13(1):89–94. 10.1016/S1473-3099(12)70148-2 . 23084831

[12] Wilson AL, Dhiman RC, Kitron U, Scott TW, van den Berg H, Lindsay SW. Benefit of insecticide-treated nets, curtains and screening on vector borne diseases, excluding malaria: a systematic review and meta-analysis. PLoS Negl Trop Dis. 2014;8(10):e3228 10.1371/journal.pntd.0003228 25299481 PMC4191944

[13] WHO. Global plan for insecticide resistance management in malaria vectors (GPIRM). Geneva: WHO; 2012.

[14] Mathenge EM, Gimnig JE, Kolczak M, Ombok M, Irungu LW, Hawley WA. Effect of permethrin-impregnated nets on exiting behavior, blood feeding success, and time of feeding of malaria mosquitoes (Diptera: Culicidae) in western Kenya. J Med Entomol. 2001;38(4):531–6. . 11476333 10.1603/0022-2585-38.4.531

[15] Kelly-Hope L, Ranson H, Hemingway J. Lessons from the past: managing insecticide resistance in malaria control and eradication programmes. Lancet Infect Dis. 2008;8(6):387–9. 10.1016/S1473-3099(08)70045-8 . 18374633

[16] Fillinger U, Lindsay SW. Larval source management for malaria control in Africa: myths and reality. Malar J. 2011;10(353):353 10.1186/1475-2875-10-353 22166144 PMC3273449

[17] Nishiura JT, Ho P, Ray K. Methoprene interferes with mosquito midgut remodeling during metamorphosis. J Med Entomol. 2003;40(4):498–507. . 14680117 10.1603/0022-2585-40.4.498

[18] Becker N, Ascher K. The use of *Bacillus thuringiensis* subsp. *israelensis* (*Bti*) against mosquitoes, with special emphasis on the ecological impact. Israel Journal of Entomology. 1998;32:63–9.

[19] Charles JF, Nielsen-LeRoux C. Mosquitocidal bacterial toxins: diversity, mode of action and resistance phenomena. Mem Inst Oswaldo Cruz. 2000;95 Suppl 1:201–6. . 11142715 10.1590/s0074-02762000000700034

[20] Scholte EJ, Knols BG, Samson RA, Takken W. Entomopathogenic fungi for mosquito control: a review. J Insect Sci. 2004;4(1):19 15861235 10.1093/jis/4.1.19 PMC528879

[21] Boyce R, Lenhart A, Kroeger A, Velayudhan R, Roberts B, Horstick O. *Bacillus thuringiensis israelensis* (*Bti*) for the control of dengue vectors: systematic literature review. Trop Med Int Health. 2013;18(5):564–77. 10.1111/tmi.12087 . 23527785

[22] Utzinger J, Tozan Y, Singer BH. Efficacy and cost-effectiveness of environmental management for malaria control. Trop Med Int Health. 2001;6(9):677–87. . 11555434 10.1046/j.1365-3156.2001.00769.x

[23] Killeen GF, Fillinger U, Kiche I, Gouagna LC, Knols BG. Eradication of *Anopheles gambiae* from Brazil: lessons for malaria control in Africa? Lancet Infect Dis. 2002;2(10):618–27. 10.1016/s1473-3099(02)00397-3 . 12383612

[24] Seyoum A, Abate D. Larvicidal efficacy of *Bacillus thuringiensis* var. *israelensis* and *Bacillus sphaericus* on *Anopheles arabiensis* in Ethiopia. World Journal of Microbiology and Biotechnology. 1997;13(1):21–4.

[25] von Hirsch H, Becker B. Cost-benefit analysis of mosquito control operations based on microbial control agents in the upper Rhine valley (Germany). J Euro Mosq Control Assoc. 2009;27:47–55.

[26] Griego V, Spence K. Inactivation of *Bacillus thuringiensis* spores by ultraviolet and visible light. Applied and Environmental Microbiology. 1978;35(5):906–10. 655707 10.1128/aem.35.5.906-910.1978 PMC242951

[27] Myasnik M, Manasherob R, Ben-Dov E, Zaritsky A, Margalith Y, Barak Ze. Comparative sensitivity to UV-B radiation of two *Bacillus thuringiensis* subspecies and other *Bacillus sp*. Current microbiology. 2001;43(2):140–3. 10.1007/s002840010276 11391479

[28] Hadapad A, Vijayalakshmi N, Hire R, Dongre T. Effect of ultraviolet radiation on spore viability and mosquitocidal activity of an indigenous ISPC-8 *Bacillus sphaericus* Neide strain. Acta tropica. 2008;107(2):113–6. 10.1016/j.actatropica.2008.04.024 18538292

[29] Karch S, Asidi N, Manzambi ZM, Salaun JJ. Efficacy of *Bacillus sphaericus* against the malaria vector *Anopheles gambiae* and other mosquitoes in swamps and rice fields in Zaire. J Am Mosq Control Assoc. 1992;8(4):376–80. . 1361940

[30] Skovmand O, Sanogo E. Experimental formulations of *Bacillus sphaericus* and *B*. *thuringiensis israelensis* against *Culex quinquefasciatus* and *Anopheles gambiae* (Diptera: Culicidae) in Burkina Faso. J Med Entomol. 1999;36(1):62–7. . 10071494 10.1093/jmedent/36.1.62

[31] Fillinger U, Knols BG, Becker N. Efficacy and efficiency of new *Bacillus thuringiensis* var *israelensis* and *Bacillus sphaericus* formulations against Afrotropical anophelines in Western Kenya. Trop Med Int Health. 2003;8(1):37–47. . 12535249 10.1046/j.1365-3156.2003.00979.x

[32] Fillinger U, Ndenga B, Githeko A, Lindsay SW. Integrated malaria vector control with microbial larvicides and insecticide-treated nets in western Kenya: a controlled trial. Bull World Health Organ. 2009;87(9):655–65. 10.2471/blt.08.055632 19784445 PMC2739910

[33] Ohana B, Margalit J, Barak Z. Fate of *Bacillus thuringiensis* subsp. *israelensis* under Simulated Field Conditions. Appl Environ Microbiol. 1987;53(4):828–31. 16347326 10.1128/aem.53.4.828-831.1987 PMC203764

[34] Fillinger U, Lindsay SW. Suppression of exposure to malaria vectors by an order of magnitude using microbial larvicides in rural Kenya. Tropical Medicine & International Health. 2006;11(11):1629–42. 17054742 10.1111/j.1365-3156.2006.01733.x

[35] Aly C, Mulla MS, Schnetter W, Xu BZ. Floating bait formulations increase effectiveness of *Bacillus thuringiensis* var. *israelensis* against *Anopheles* larvae. J Am Mosq Control Assoc. 1987;3(4):583–8. . 3504944

[36] Becker N, Petrić D, Boase C, Lane J, Zgomba M, Dahl C, et al Mosquitoes and their control: Springer; 2010.

[37] Logan JG, Birkett MA. Semiochemicals for biting fly control: their identification and exploitation. Pest management science. 2007;63(7):647–57. 10.1002/ps.1408 17549674

[38] Logan JG, Pickett JA, Cameron MM. 6 Vector Control Using Semiochemicals Biological and Environmental Control of Disease Vectors. 2013:95.

[39] Michaelakis A, Mihou AP, Koliopoulos G, Couladouros EA. Attract-and-kill strategy. Laboratory studies on hatched larvae of *Culex pipiens*. Pest Manag Sci. 2007;63(10):954–9. 10.1002/ps.1418 . 17708518

[40] Vale G, Hall D. The use of 1-octen-3-ol, acetone and carbon dioxide to improve baits for tsetse flies, Glossina spp.(Diptera: Glossinidae). Bulletin of Entomological Research. 1985;75(02):219–32.

[41] Vale G, Hall D. The role of 1-octen-3-ol, acetone and carbon dioxide in the attraction of tsetse flies, *Glossina spp*. (Diptera: Glossinidae), to ox odour. Bulletin of Entomological Research. 1985;75(02):209–18.

[42] Muirhead-Thompson R. Trap responses of flying insects: the influence of trap design on capture efficiency. London: Academic Press; 1991.

[43] Millar JG. Pheromones of true bugs The Chemistry of Pheromones and Other Semiochemicals II: Springer; 2005 p. 37–84.

[44] Logan JG, Seal NJ, Cook JI, Stanczyk NM, Birkett MA, Clark SJ, et al Identification of Human-Derived Volatile Chemicals That Interfere With Attraction of the Scottish Biting Midge and Their Potential Use as Repellents. Journal of Medical Entomology. 2009;46(2):208–19. 10.1603/033.046.0205 19351071

[45] Afify A, Galizia CG. Chemosensory Cues for Mosquito Oviposition Site Selection. J Med Entomol. 2015;52(2):120–30. 10.1093/jme/tju024 . 26336295

[46] Osgood CE. An oviposition pheromone associated with the egg rafts of *Culex tarsalis*. J Econ Entomol. 1971;64(5):1038–41. . 5122321 10.1093/jee/64.5.1038

[47] Osgood CE, Kempster RH. An air-flow olfactometer for distinguishing between oviposition attractants and stimulants of mosquitoes. Journal of economic entomology. 1971;64(5):1109–10.

[48] Mboera L, Mdira K, Salum F, Takken W, Pickett J. Influence of synthetic oviposition pheromone and volatiles from soakage pits and grass infusions upon oviposition site-selection of *Culex* mosquitoes in Tanzania. Journal of Chemical Ecology. 1999;25(8):1855–65.

[49] Mboera LE, Takken W, Mdira KY, Pickett JA. Sampling gravid *Culex quinquefasciatus* (Diptera: Culicidae) in Tanzania with traps baited with synthetic oviposition pheromone and grass infusions. J Med Entomol. 2000;37(1):172–6. . 15218923 10.1603/0022-2585-37.1.172

[50] Mboera LE, Takken W, Sambu EZ. The response of *Culex quinquefasciatus* (Diptera: culicidae) to traps baited with carbon dioxide, 1-octen-3-ol, acetone, butyric acid and human foot odour in Tanzania. Bull Entomol Res. 2000;90(2):155–9. . 10948375 10.1017/s0007485300000262

[51] Sullivan GA, Liu C, Syed Z. Oviposition signals and their neuroethological correlates in the *Culex pipiens* complex. Infect Genet Evol. 2014;28:735–43. 10.1016/j.meegid.2014.10.007 . 25460826

[52] Atterholt C, Delwiche M, Rice R, Krochta J. Study of biopolymers and paraffin as potential controlled-release carriers for insect pheromones. Journal of Agricultural and Food Chemistry. 1998;46(10):4429–34.

[53] Atterholt CA, Delwiche MJ, Rice RE, Krochta JM. Controlled release of insect sex pheromones from paraffin wax and emulsions. J Control Release. 1999;57(3):233–47. . 9895411 10.1016/s0168-3659(98)00119-9

[54] Atlas RM. Microbial degradation of petroleum hydrocarbons: an environmental perspective. Microbiological reviews. 1981;45(1):180 7012571 10.1128/mr.45.1.180-209.1981 PMC281502

[55] Leahy JG, Colwell RR. Microbial degradation of hydrocarbons in the environment. Microbiological reviews. 1990;54(3):305–15. 2215423 10.1128/mr.54.3.305-315.1990 PMC372779

[56] Laurence BR, Mori K, Otsuka T, Pickett JA, Wadhams LJ. Absolute configuration of mosquito oviposition attractant pheromone, 6-acetoxy-5-hexadecanolide. J Chem Ecol. 1985;11(5):643–8. 10.1007/BF00988573 . 24310128

[57] Laurence BR, Pickett JA. Erythro-6-acetoxy-5-hexadecanolide, the major component of a mosquito oviposition attractant pheromone. Journal of the Chemical Society, Chemical Communications. 1982;(1):59–60.

[58] Kitau J, Pates H, Rwegoshora TR, Rwegoshora D, Matowo J, Kweka EJ, et al The effect of Mosquito Magnet^®^ Liberty Plus trap on the human mosquito biting rate under semi-field conditions. Journal of the American Mosquito Control Association. 2010;26(3):287–94. 10.2987/09-5979.1 21033055

[59] Mulla MS, Su T, Thavara U, Tawatsin A, Ngamsuk W, Pan-Urai P. Efficacy of new formulations of the microbial larvicide *Bacillus sphaericus* against polluted water mosquitoes in Thailand. J Vector Ecol. 1999;24(1):99–110. . 10436884

[60] Gunasekaran K, Shriram AN, Elangovan A, Narayanan RJ, Balaraman K. Efficacy of *Bacillus sphaericus* in different breeding habitats of *Culex quinquefasciatus*. Southeast Asian J Trop Med Public Health. 1996;27(3):622–7. . 9185281

[61] Barbazan P, Baldet T, Darriet F, Escaffre H, Djoda DH, Hougard JM. Control of *Culex quinquefasciatus* (Diptera: Culicidae) with *Bacillus sphaericus* in Maroua, Cameroon. J Am Mosq Control Assoc. 1997;13(3):263–9. . 9383769

[62] Sharma SK, Upadhyay AK, Haque MA, Raghavendra K, Dash AP. Field evaluation of a previously untested strain of biolarvicide (*Bacillus thuringiensis israelensis* H14) for mosquito control in an urban area of Orissa, India. J Am Mosq Control Assoc. 2008;24(3):410–4. 10.2987/5704.1 . 18939694

[63] Dambach P, Louis VR, Kaiser A, Ouedraogo S, Sie A, Sauerborn R, et al Efficacy of *Bacillus thuringiensis* var. *israelensis* against malaria mosquitoes in northwestern Burkina Faso. Parasit Vectors. 2014;7:371 10.1186/1756-3305-7-371 25128297 PMC4262221

[64] Dambach P, Traore I, Becker N, Kaiser A, Sie A, Sauerborn R. EMIRA: Ecologic Malaria Reduction for Africa—innovative tools for integrated malaria control. Glob Health Action. 2014;7:25908 10.3402/gha.v7.25908 25377345 PMC4223283

[65] Stone CM, Lindsay SW, Chitnis N. How effective is integrated vector management against malaria and lymphatic filariasis where the diseases are transmitted by the same vector? PLoS Negl Trop Dis. 2014;8(12):e3393 10.1371/journal.pntd.0003393 25501002 PMC4263402

[66] Fillinger U, Kannady K, William G, Vanek MJ, Dongus S, Nyika D, et al A tool box for operational mosquito larval control: preliminary results and early lessons from the Urban Malaria Control Programme in Dar es Salaam, Tanzania. Malar J. 2008;7(1):20 10.1186/1475-2875-7-20 18218148 PMC2259364

[67] Majambere S, Pinder M, Fillinger U, Ameh D, Conway DJ, Green C, et al Is mosquito larval source management appropriate for reducing malaria in areas of extensive flooding in The Gambia? A cross-over intervention trial. Am J Trop Med Hyg. 2010;82(2):176–84. 10.4269/ajtmh.2010.09-0373 20133989 PMC2813154

[68] Skovmand O, Ouedraogo TDA, Sanogo E, Samuelsen H, Toé LP, Bosselmann R, et al Cost of Integrated Vector Control With Improved Sanitation and Road Infrastructure Coupled With the Use of Slow-Release *Bacillus sphaericus* Granules in a Tropical Urban Setting. Journal of Medical Entomology. 2011;48(4):813–21. 10.1603/me10041 21845940

[69] Jones CM, Machin C, Mohammed K, Majambere S, Ali AS, Khatib BO, et al Insecticide resistance in *Culex quinquefasciatus* from Zanzibar: implications for vector control programmes. Parasit Vectors. 2012;5:78 10.1186/1756-3305-5-78 22520274 PMC3349604

[70] WHO. Lymphatic filariasis: a handbook of practical entomology for national lymphatic filariasis elimination programmes: WHO; 2013.

[71] Maheu-Giroux M, Castro MC. Cost-effectiveness of larviciding for urban malaria control in Tanzania. Malar J. 2014;13:477 10.1186/1475-2875-13-477 25476586 PMC4289051

[72] Kar I, Eapen A, Ravindran KJ, Chandrahas RK, Appavoo NC, Sadanand AV, et al Field evaluation of *Bacillus sphaericus*, H5a5b and *B*. *thuringiensis* var. *israelensis*, H-14 against the Bancroftian filariasis vector *Culex quinquefasciatus*, Say in Chennai, India. Indian J Malariol. 1997;34(1):25–36. . 9291671

[73] Shililu JI, Tewolde GM, Brantly E, Githure JI, Mbogo CM, Beier JC, et al Efficacy of *Bacillus thuringiensis israelensis*, *Bacillus sphaericus* and temephos for managing *Anopheles* larvae in Eritrea. J Am Mosq Control Assoc. 2003;19(3):251–8. . 14524547

[74] Wirth MC, Park HW, Walton WE, Federici BA. Cyt1A of *Bacillus thuringiensis* delays evolution of resistance to Cry11A in the mosquito *Culex quinquefasciatus*. Appl Environ Microbiol. 2005;71(1):185–9. 10.1128/AEM.71.1.185-189.2005 15640186 PMC544219

[75] Wirth MC, Walton WE, Federici BA. Evolution of resistance to the *Bacillus sphaericus* Bin toxin is phenotypically masked by combination with the mosquitocidal proteins of *Bacillus thuringiensis* subspecies israelensis. Environ Microbiol. 2010;12(5):1154–60. 10.1111/j.1462-2920.2010.02156.x . 20141526

[76] Mafra-Neto A, De Lame F, Fettig CJ, Munson AS, Perring TM, Stelinski LL, et al Manipulation of insect behavior with specialized pheromone and lure application technology (SPLAT^®^). Pest Management with Natural Products. 2013;1141:31–58.

[77] Mafra-Neto A, Fettig CJ, Munson AS, Rodriguez-Saona C, Holdcraft R, Faleiro JR, et al Development of specialized pheromone and lure application technologies (SPLAT^®^) for management of coleopteran pests in agricultural and forest systems. Biopesticides: State of the Art and Future Opportunities. 2014;1172:211–42.

[78] Stelinski L, Lapointe S, Meyer W. Season—long mating disruption of citrus leafminer, *Phyllocnistis citrella* Stainton, with an emulsified wax formulation of pheromone. Journal of Applied Entomology. 2010;134(6):512–20.

[79] Stelinski LL, Miller JR, Ledebuhr R, Siegert P, Gut LJ. Season-long mating disruption of *Grapholita molesta* (Lepidoptera: Tortricidae) by one machine application of pheromone in wax drops (SPLAT-OFM). Journal of Pest Science. 2007;80(2):109–17. 10.1007/s10340-007-0162-0

[80] Clements A. The biology of mosquitoes Vol. 2 Sensory reception and behaviour. CABI, Wallinford 1999.

[81] Laurence B, Pickett J. An oviposition attractant pheromone in *Culex quinquefasciatus* Say (Diptera: Culicidae). Bulletin of Entomological Research. 1985;75(02):283–90.

[82] Hwang YS, Mulla MS, Chaney JD, Lin GG, Xu HJ. Attractancy and species specificity of 6-acetoxy-5-hexadecanolide, a mosquito oviposition attractant pheromone. J Chem Ecol. 1987;13(2):245–52. 10.1007/BF01025885 . 24301804

[83] Olagbemiro TO, Birkett MA, Mordue AJ, Pickett JA. Laboratory and field responses of the mosquito, *Culex quinquefasciatus*, to plant-derived *Culex* spp. oviposition pheromone and the oviposition cue skatole. Journal of chemical ecology. 2004;30(5):965–76. 15274442 10.1023/b:joec.0000028461.86243.19

[84] Barbosa RM, Souto A, Eiras AE, Regis L. Laboratory and field evaluation of an oviposition trap for *Culex quinquefasciatus* (Diptera: Culicidae). Mem Inst Oswaldo Cruz. 2007;102(4):523–9. . 17612774 10.1590/s0074-02762007005000058

[85] Braks MA, Leal WS, Carde RT. Oviposition responses of gravid female *Culex quinquefasciatus* to egg rafts and low doses of oviposition pheromone under semifield conditions. J Chem Ecol. 2007;33(3):567–78. 10.1007/s10886-006-9223-8 . 17252215

[86] Leal WS, Barbosa RM, Xu W, Ishida Y, Syed Z, Latte N, et al Reverse and conventional chemical ecology approaches for the development of oviposition attractants for *Culex* mosquitoes. PLoS One. 2008;3(8):e3045 10.1371/journal.pone.0003045 18725946 PMC2516325

[87] Wachira SW, Ndung'u M, Njagi PG, Hassanali A. Comparative responses of ovipositing *Anopheles* gambiae and *Culex quinquefasciatus* females to the presence of *Culex* egg rafts and larvae. Med Vet Entomol. 2010;24(4):369–74. 10.1111/j.1365-2915.2010.00913.x . 21058965

[88] Lapointe SL, Stelinski LL. An applicator for high viscosity semiochemical products and intentional treatment gaps for mating disruption of *Phyllocnistis citrella*. Entomologia Experimentalis et Applicata. 2011;141(2):145–53. 10.1111/j.1570-7458.2011.01178.x

[89] Angelon KA, Petranka JW. Chemicals of predatory mosquitofish (*Gambusia affinis*) influence selection of oviposition site by *Culex* mosquitoes. J Chem Ecol. 2002;28(4):797–806. . 12035927 10.1023/a:1015292827514

[90] Mokany A, Shine R. Oviposition site selection by mosquitoes is affected by cues from conspecific larvae and anuran tadpoles. Austral Ecology. 2003;28(1):33–7.

[91] Blaustein L, Blaustein J, Chase J. Chemical detection of the predator *Notonecta irrorata* by ovipositing *Culex* mosquitoes. J Vector Ecol. 2005;30(2):299–301. . 16599167

[92] Van Dam AR, Walton WE. The effect of predatory fish exudates on the ovipostional behaviour of three mosquito species: *Culex quinquefasciatus*, *Aedes aegypti* and *Culex tarsalis*. Med Vet Entomol. 2008;22(4):399–404. 10.1111/j.1365-2915.2008.00764.x . 19120968

[93] Siegwart M, Graillot B, Blachere Lopez C, Besse S, Bardin M, Nicot PC, et al Resistance to bio-insecticides or how to enhance their sustainability: a review. Front Plant Sci. 2015;6:381 10.3389/fpls.2015.00381 26150820 PMC4472983

[94] Yuan Z, Zhang Y, Cai Q, Liu E-Y. High-level field resistance to *Bacillus sphaericus* C3-41 in *Culex quinquefasciatus* from Southern China. Biocontrol Science and Technology. 2000;10(1):41–9.

[95] Zahiri NS, Su T, Mulla MS. Strategies for the management of resistance in mosquitoes to the microbial control agent *Bacillus sphaericus*. J Med Entomol. 2002;39(3):513–20. . 12061449 10.1603/0022-2585-39.3.513

[96] Mulla MS, Thavara U, Tawatsin A, Chomposri J, Su T. Emergence of resistance and resistance management in field populations of tropical *Culex quinquefasciatus* to the microbial control agent *Bacillus sphaericus*. J Am Mosq Control Assoc. 2003;19(1):39–46. . 12674533

[97] Suckling DM, Brockerhoff EG, Stringer LD, Butler RC, Campbell DM, Mosser LK, et al Communication Disruption of *Epiphyas postvittana* (Lepidoptera: Tortricidae) By Using Two Formulations at Four Point Source Densities in Vineyards. Journal of Economic Entomology. 2012;105(5):1694–701. 10.1603/ec12130 23156166

[98] Lapointe SL, Stelinski LL, Keathley CP, Mafra-Neto A. Intentional Coverage Gaps Reduce Cost of Mating Disruption for *Phyllocnistis citrella* (Lepidoptera: Gracillariidae) in Citrus. Journal of Economic Entomology. 2014;107(2):718–26. 10.1603/ec13388 24772554

[99] Vargas RI, Piñero JC, Mau RFL, Stark JD, Hertlein M, Mafra-Neto A, et al Attraction and mortality of oriental fruit flies to SPLAT-MAT-methyl eugenol with spinosad. Entomologia Experimentalis et Applicata. 2009;131(3):286–93. 10.1111/j.1570-7458.2009.00853.x

[100] El-Shafie HAF, Faleiro JR, Al-Abbad AH, Stoltman L, Mafra-Neto A. Bait-Free Attract and Kill Technology (Hook^™^ RPW) to Suppress Red Palm Weevil, *Rhynchophorus ferrugineus* (Coleoptera: Curculionidae) in Date Palm*. Florida Entomologist. 2011;94(4):774–8. 10.1653/024.094.0407

[101] Land M, Miljand M. Biological control of mosquitoes using Bacillus thuringiensis israelensis: a pilot study of effects on target organisms, non-target organisms and humans. Mistra EviEM, Stockholm, Sweden 2014.

[102] Rinker DC, Pitts RJ, Zhou X, Suh E, Rokas A, Zwiebel LJ. Blood meal-induced changes to antennal transcriptome profiles reveal shifts in odor sensitivities in *Anopheles gambiae*. Proc Natl Acad Sci U S A. 2013;110(20):8260–5. 10.1073/pnas.1302562110 23630291 PMC3657813

[103] Lindh JM, Okal MN, Herrera-Varela M, Borg-Karlson AK, Torto B, Lindsay SW, et al Discovery of an oviposition attractant for gravid malaria vectors of the *Anopheles gambiae* species complex. Malar J. 2015;14(1):119 10.1186/s12936-015-0636-0 25885703 PMC4404675

[104] Ong S-Q, Jaal Z. Investigation of mosquito oviposition pheromone as lethal lure for the control of *Aedes aegypti* (L.)(Diptera: Culicidae). Parasites & vectors. 2015;8(1):1–7. 25588346 10.1186/s13071-015-0639-2 PMC4299678

[105] Fenwick A. The global burden of neglected tropical diseases. Public Health. 2012;126(3):233–6. 10.1016/j.puhe.2011.11.015 . 22325616

[106] Hotez PJ. Ten global "hotspots" for the neglected tropical diseases. PLoS Negl Trop Dis. 2014;8(5):e2496 10.1371/journal.pntd.0002496 24873825 PMC4038631

[107] Hotez PJ, Alvarado M, Basanez MG, Bolliger I, Bourne R, Boussinesq M, et al The global burden of disease study 2010: interpretation and implications for the neglected tropical diseases. PLoS Negl Trop Dis. 2014;8(7):e2865 10.1371/journal.pntd.0002865 25058013 PMC4109880

